# Supplementary figures and images for: Implications of diffusion and time-varying morphogen gradients for the dynamic positioning and precision of bistable gene expression boundaries
Source: PLoS Comput Biol. 2021 Jun 1;17(6):e1008589. doi: 10.1371/journal.pcbi.1008589 (PMC8195430; doi:10.1371/journal.pcbi.1008589)

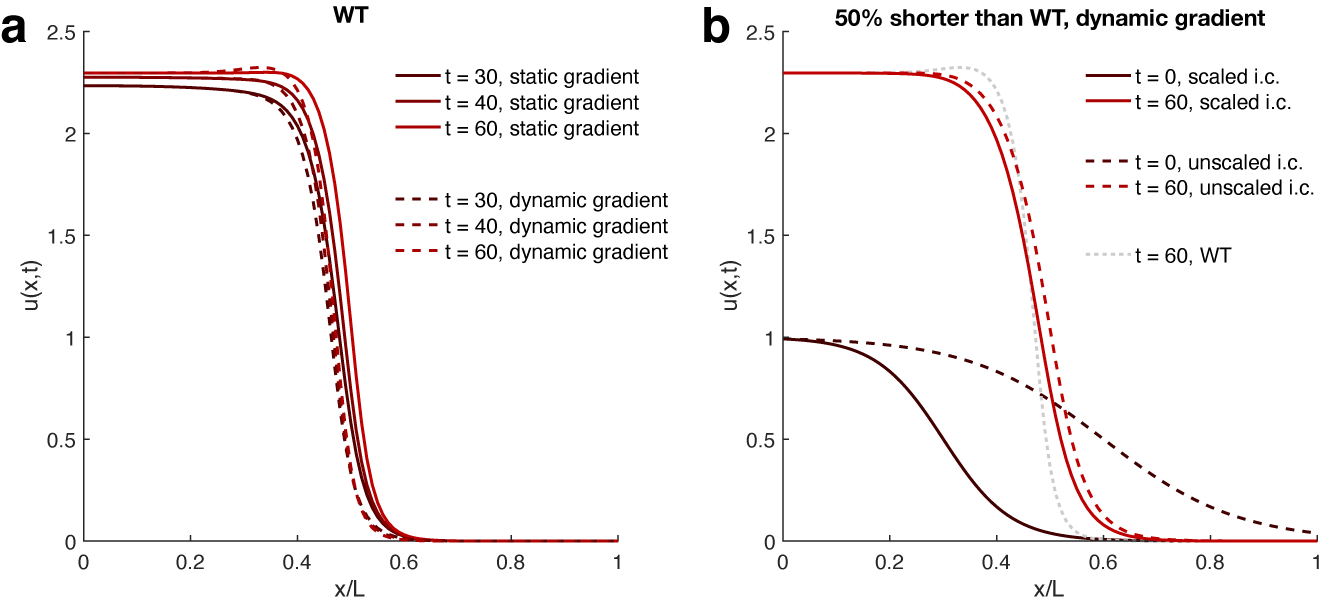

Supplement: S1 Fig — (TIF) [file pcbi.1008589.s002.tif]
