# Supplementary material for: Implications of diffusion and time-varying morphogen gradients for the dynamic positioning and precision of bistable gene expression boundaries
Source: PLoS Comput Biol. 2021 Jun 1;17(6):e1008589. doi: 10.1371/journal.pcbi.1008589 (PMC8195430; doi:10.1371/journal.pcbi.1008589)
Supplement: S1 Appendix — (PDF) [file pcbi.1008589.s003.pdf]

S1 Appendix

2D autonomous model for precision enhancement

In the following discussion, we consider the 1D time-dependent (nonautonomous) model

$$\frac{du(t,x)}{dt} = (\alpha(t,x) - b(x)) + u(t,x) - u(t,x)^3 \tag{1}$$

at coordinates x and times t. We assume there is some set of coordinates where the system is instantaneously bistable, with boundaries denoted by $x_1(t), x_2(t)$; that is, $x < x_1(t)$ implies (1) is instantaneously monostable (high U) at time t, $x_1(t) \leq x \leq x_2(t)$ implies (1) is instantaneously bistable at time t, and $x_2(t) < x$ implies (1) is instantaneously monostable (low U). Assume several realizations of the process are given, each ultimately representing a horizontal slice of a 2D "embryo". For convenience we define $\mathcal{X}_{high}(t) = \{x : x < x_1(t)\}$, $\mathcal{X}_{bi}(t) = \{x : x_1(t) < x < x_2(t)\}$, $\mathcal{X}_{low}(t) = \{x : x_2(t) < x(t)\}$, where the subscripts are derived with respect to the expected steady-state concentration of U.

We will consider the case where $\alpha(t,x)$ varies smoothly and monotonically in time, that is, $t_2 > t_1 \implies \alpha(t_2,x) \leq \alpha(t_1,x)\ \forall x$, and asympototically approaches a finite value: $\alpha(t \to \infty, x) \to \alpha^*(x)$. For most of this discussion we will assume $\alpha(t,x)$ decreases, although it is conceptually straightforward to generalize our discussion to the case where $\alpha(t,x)$ increases. For our setup, decrease of $\alpha(t,x)$ in time means $t_2 > t_1 \implies x_1(t_2) < x_1(t_1)$ and $x_2(t_2) < x_2(t_1)$. We will further assume that $\mathcal{X}_{bi}(\infty) \subset \mathcal{X}_{high}(0)$ and $\mathcal{X}_{bi}(0) \subset \mathcal{X}_{low}(\infty)$, although this requirement may be relaxed.

If the temporal evolution of $\alpha(t,x)$ is much faster than the dynamics of u, then the behavior of the system is well approximated by (1) with $\alpha(x) = \alpha^*(x)$. If the dynamics of $u(t,x)$ are much faster than the dynamics of $\alpha(t,x)$ and the system satisfies certain technical requirements including slow evolution of $\alpha(t,x)$ [1], then we expect that $u(t,x)$ will rapidly converge to the instantaneous system attractors and will in effect traverse the *stable paths* of those attractors. Thus, $x \in \mathcal{X}_{high}(0) \implies u(\infty,x)$ high, and $x \in \mathcal{X}_{low}(\infty) \implies u(\infty,x)$ low regardless of initial condition. In other words, at infinite time there should be no remaining irregularity at the boundary between high and low U.

If the dynamics are on similar timescales, however, then variation in initial conditions may exert influence on system outcome: Two trajectories $u(t,x)$ with $x \in \mathcal{X}_{bi}(\infty) \subset \mathcal{X}_{high}(0)$ may converge to different steady states, such that the final boundary is imprecise. Guaranteeing a precise boundary, therefore, becomes a matter of restricting variation in initial conditions for $x \in \mathcal{X}_{bi}(\infty)$ to the basin of attraction of the stable path corresponding to high U.

To examine this question intuitively, we now assume a particular form of $\alpha(x,t)$ that allows us to rewrite (1) as a time-independent (autonomous) system. In particular, suppose that the morphogen at coordinate x decreases in concentration exponentially with rate r:

$$\alpha(t) = \alpha(0)e^{-rt}$$

where for simplicity of notation we have dropped the dependence of α on x. The time evolution of $\alpha(t)$ may be described by

$$\frac{d\alpha(t)}{dt} = -r\alpha(t)$$

for initial condition $\alpha(0)$. We can then rewrite (1) as

$$\begin{cases} \frac{d\alpha(t)}{dt} = -r\alpha(t) \\ \frac{du(t)}{dt} = (\alpha(t) - b) + u(t) - u(t)^3 \end{cases} \tag{2}$$

where $\alpha(0) \in \mathbb{R}_+ \implies \alpha(t) \in \mathbb{R}_+ \ \forall t$, $b \in \mathbb{R}$, and we have again dropped the dependence of u and b on x for simplicity of notation. The system (2) has saddle-node bifurcations at each of $(\alpha - b) = \pm\frac{2}{3}\sqrt{\frac{1}{3}}$ [2]. Since $\alpha(t \to \infty) \to 0$, the steady states in this system are set by b:

- one stable high state if $b < -\frac{2}{3}\sqrt{\frac{1}{3}}$,
- two stable states and one intermediate unstable state if $-\frac{2}{3}\sqrt{\frac{1}{3}} < b < \frac{2}{3}\sqrt{\frac{1}{3}}$,
- one stable low state if $b > \frac{2}{3}\sqrt{\frac{1}{3}}$.

In a toy model of an embryo, we would expect r to remain constant across the axis while $\alpha(0)$ and b form spatial gradients determining the initial morphogen concentration and mono-/bistability respectively. Since monostable systems have outcomes that do not depend on initial conditions, to ensure boundary regularity we must ensure that bistable cells end up in the high state. Specifically, we seek to determine for which range of initial morphogen concentrations $\alpha(0)$ and chemical concentrations $u(0)$ trajectories terminate in the high state. This is the definition of the basin of attraction for the high state, represented in Fig 1 by the region above the thick red line in each plot. Decreasing b or r increases the size of the basin of attraction for the high state, which matches the intuition that a longer time spent with a higher input ought to bias the system toward a higher state. As r increases, the system behaves effectively as though $\alpha(t) = 0$, since trajectories evolve much more slowly than the change in the input level.

Convergence rate to steady state

The convergence rate to a stable steady state $u^* = (u_1^*, u_2^*)$ at coordinate x is determined by the eigenvalues λ_i of the Jacobian $J(u^*)$ of Eq (3) in the main text for $\alpha(x) = be^{-\frac{x}{\lambda}}$, as

$$\sigma_{u^*}(x) = \min_i \left| \Re \left\{ \lambda_i \left(J_x(u^*) \right) \right\} \right|, \tag{3}$$

where $\Re$ denotes the real part of the argument. Then

$$\sigma = \min_x \min_{u^*} \sigma_{u^*}(x) \tag{4}$$

where the second minimum is taken across the set of stable steady states existing at coordinate x. For the model with a static gradient considered in Eq (3) in the main text, the eigenvalues are analytically determined to be

$$\lambda_i(J_x(u^*)) = \frac{(f_1(x) + f_2) \pm \sqrt{(f_1(x) - f_2)^2 - 4\beta^2}}{2} \tag{5}$$

where $f_1(x) = h_a(\alpha_\infty(x))a_1 h_r'(u_2^*)$, $f_2 = a_2 h' r(u_1^*)$, and

$$h_r'(y) = \frac{dh_r}{dy} = \frac{-\frac{n_r}{K_r}\left(\frac{y}{K_r}\right)^{n_r - 1}}{\left(1 + \left(\frac{y}{K_r}\right)^{n_r}\right)^2}. \tag{6}$$

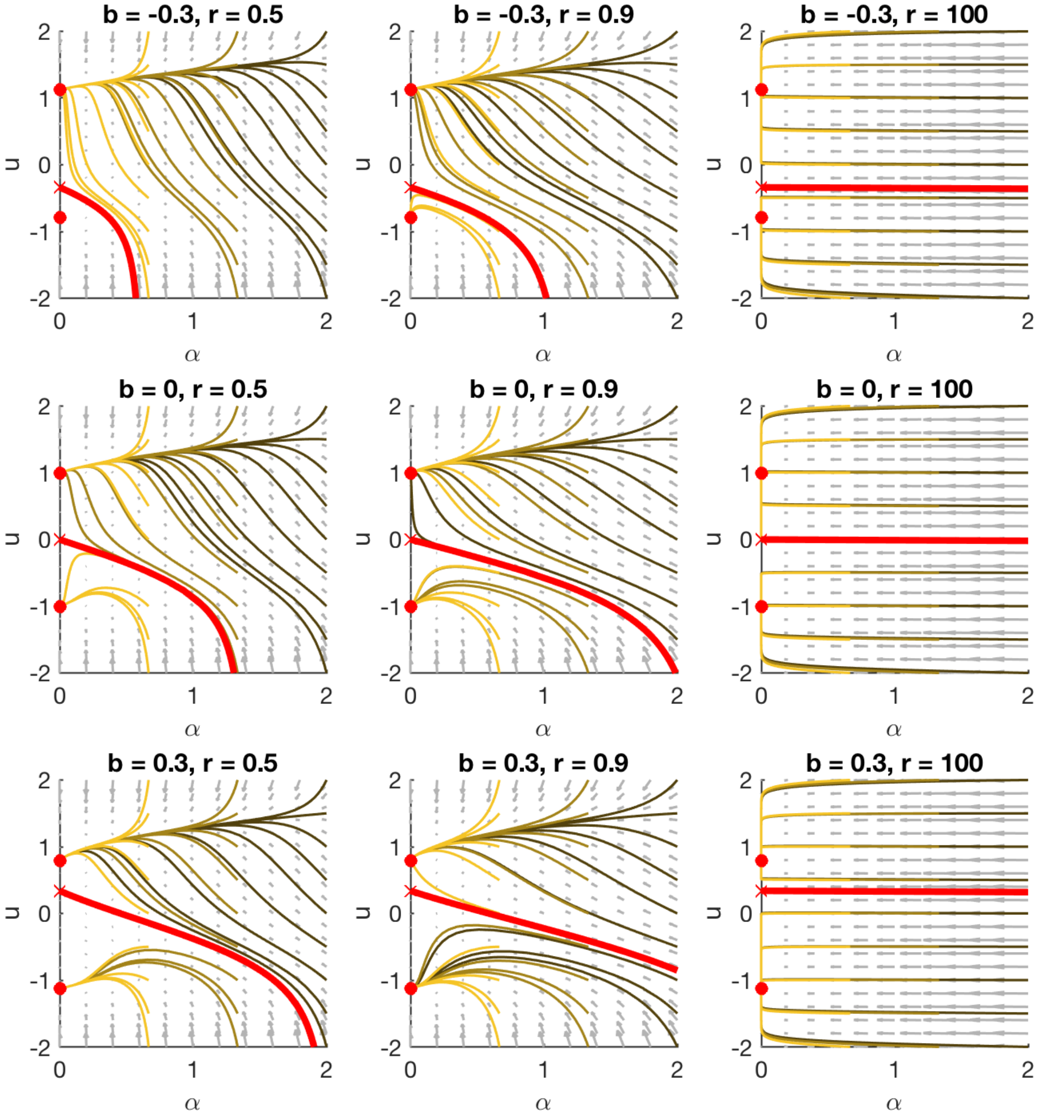

Figure 1: **Slowing the rate of decrease in morphogen concentration causes more trajectories to reach the high steady state.** Phase spaces (gray vector field) overlaid with sample trajectories (gold) for (2) with varying b, r. Darker trajectories start with higher $\alpha(0)$. Red circles denote stable stady states and the red x denotes the unstable steady state. The thick red line is the separatrix, so named because it separates initial conditions that end in the high state (above the line) from those that end in the low state (below the line). As b or r is decreased (to the top or left), the basin of attraction of the high state increases in size; i.e., trajectories starting with a wider range of initial conditions will end up in the high state. For fixed b, r, a boundary can form when the initial gradient $\alpha(0)$ spans the separatrix.

References

[1] Ashwin P, Perryman C, Wieczorek S. Parameter shifts for nonautonomous systems in low dimension: bifurcation- and rate-induced tipping. Nonlinearity. 2017;30. doi:10.1088/1361-6544/aa675b.

[2] Strogatz SH. Nonlinear dynamics and chaos: with applications to physics, biology, chemistry, and engineering. Addison-Wesley Publishing Company;.
