# Supplementary material for: Implications of diffusion and time-varying morphogen gradients for the dynamic positioning and precision of bistable gene expression boundaries
Source: PLoS Comput Biol. 2021 Jun 1;17(6):e1008589. doi: 10.1371/journal.pcbi.1008589 (PMC8195430; doi:10.1371/journal.pcbi.1008589)
Supplement: S2 Appendix — (PDF) [file pcbi.1008589.s004.pdf]

S2 Appendix: 1D model for front localization on a finite domain

Here we provide a more mathematical explanation for bistable front localization using a single-species model in a one-dimensional reaction domain. We consider systems of the form

$$\frac{\partial u(t,x)}{\partial t} = f(u(t,x)) + D\frac{\partial^2 u(t,x)}{\partial x^2} \tag{1}$$

with $u(t,x) \in \mathbb{R}$ and

$$f(u) = au(u-1)(\bar{\alpha} - u) \tag{2}$$

for $0 < \bar{\alpha} < 1$. We assume $u(t,x) \in [0,1]\ \forall (t,x)$.

Suppose $x \in \mathbb{R}$, such that the reaction domain is an infinite line. Then the system (1) admits a traveling wavefront solution with profile $U(x + \bar{c}t) = u(t,x)$. Analytical solutions are known for $f(\cdot)$ as in (2). In particular,

$$U(\xi) = \frac{1}{2}\left(1 + \tanh\left(\frac{1}{2}\sqrt{\frac{aD}{2}}\xi\right)\right) \tag{3}$$

and

$$\bar{c} = \sqrt{\frac{aD}{2}}\,(1 - 2\bar{\alpha}) \tag{4}$$

such that the wave ceases to propagate when $\bar{\alpha} = \frac{1}{2}$ [1]. (Traveling fronts on infinite discrete lattices may localize for a wider range of parameters than corresponding continuous systems, depending, for example, on coupling strength [2] or propagation direction [3].)

Now consider the solution to (1) on a finite domain $x \in [x_0, x_1]$ with no-flux (Neumann) boundary conditions

$$\left.\frac{\partial u(t,x)}{\partial t}\right|_{x=x_0} = \left.\frac{\partial u(t,x)}{\partial t}\right|_{x=x_1} = 0. \tag{5}$$

Although no stable, nonhomogeneous steady-state solutions exist for $\bar{\alpha} \neq \frac{1}{2}$ in the continuous case [4, 5, 6], transient (but potentially long-lived) wavefronts may emerge that propogate from one end of the domain to the other before vanishing [7, 8].

Researchers have previously noted that spatial variation in 2D domain shape [9] or reaction-diffusion terms (e.g., [10, 11]) can lead to stable nonhomogeneous solutions. We now introduce a class of spatial inhomogeneities that stabilize particular solutions comprising one or more localized fronts, where by "front" we mean an approximately sigmoidal curve. Let

$$\frac{\partial u(t,x)}{\partial t} = f(u(t,x),x) + D\frac{\partial^2 u(t,x)}{\partial x^2} \tag{6}$$

where

$$f(u(t,x),x) = au(t,x)(u(t,x)-1)(\alpha(x) - u(t,x)). \tag{7}$$

The dependence of f on x permits stable nonhomogeneous solutions to exist. We enforce that $\alpha(x) \in (0,1)\ \forall x$, such that $f \in [0,a]$. We further assume $\alpha(x)$ is smooth and monotonically decreasing, i.e., $\alpha(x_0) > \frac{1}{2}$, $\alpha(x_1) < \frac{1}{2}$, and $\alpha(x_l) = \frac{1}{2}$ for some $x_l \in (x_0, x_1)$. We associate with each $x^* \in [x_0, x_1]$ a local front velocity $c(x^*)$, which is the velocity (4) of a monotonically increasing traveling wavefront solution to (1) for $\bar{\alpha} = \alpha(x^*)$.

Suppose $u(x,0)$ is a monotonically increasing front with transition width much smaller than the length of the domain. Since $\alpha(x)$ is defined such that $c(x) < 0$ for $x < x_l$ (rightward propagation) and $c(x) > 0$ for $x > x_l$ (leftward propagation), we would intuitively expect the front to approach the localization point x_l where $c(x_l) = 0$ regardless of where the front is located in the domain. This observation suggests that there is a stable steady-state front solution centered at x_l, which we numerically confirmed to be a stable nonhomogeneous solution in the discrete-space case. Note that these front solutions do not match the stable profile $U(\cdot)$ for any corresponding system with homogeneous $\alpha(x) = \bar{\alpha}$. Fig 1 illustrates such a system with

$$\alpha(x) = e^{-\frac{x}{\lambda}} \tag{8}$$

where λ is half the domain length. Reducing D reduces the boundary width, steepening the transition between expression states.

A system with a localized front both admits a stable steady-state front solution and can reach that solution from spatially homogeneous initial conditions. Define for each x an equivalent system to (6) with no diffusion, governed by

$$\frac{dv(t,x)}{dt} = f(v(t,x),x) \tag{9}$$

for $f(\cdot,\cdot)$ as in (7). At each point x, $v(x,\cdot)$ has two stable steady states $v^*_{low}(x) = 0$ and $v^*_{high}(x) = 1$. Let $v(t, x = 0) = b \in (0,1)$. For a given x, b will fall in the basin of attraction of $v^*_{low}(x)$ if $b < \alpha(x)$ and in the basin of attraction of $v^*_{high}(x)$ if $b > \alpha(x)$. In the corresponding diffusive system (6), the front will appear between x_l and x_b satisfying $b = \alpha(x_b)$, with the location depending on the relative rates of front emergence (local divergence toward high or low steady state) and instantaneous front propagation. Thus, persistent spatial parameter variation produces a front with a particular orientation and ensures the convergence of that front to a fixed location in the domain, without requiring spatial bias in initial conditions (Fig 1a).

If we know the relationship of the local front velocity to points in the domain x, we can approximate the position of the boundary $w(t)$ by solving the ODE

$$\frac{dw}{dt} = c(w+\epsilon) \tag{10}$$

for some possibly nonzero adjustment parameter ϵ. For this example we will let $\epsilon = 0$. With the system (6) and $f(\cdot)$ as in (7), we have local front velocities (4)

$$\frac{dw}{dt} = \sqrt{\frac{aD}{2}}\left(1 - 2\alpha(x)\right). \tag{11}$$

For $\alpha(x)$ as in (8), (11) can be analytically solved to give

$$w(t) = \lambda \ln\left(2 + \left(e^{\frac{w(0)}{\lambda}} - 2\right) e^{-\sqrt{\frac{Da}{2\lambda^2}}t}\right). \tag{12}$$

In Fig 2 we compare the PDE simulation to the above solution to the ODE approximation. Notice that front speed increases with increasing D, but all solutions approach the same steady state where $\alpha(w) = \frac{1}{2}$.

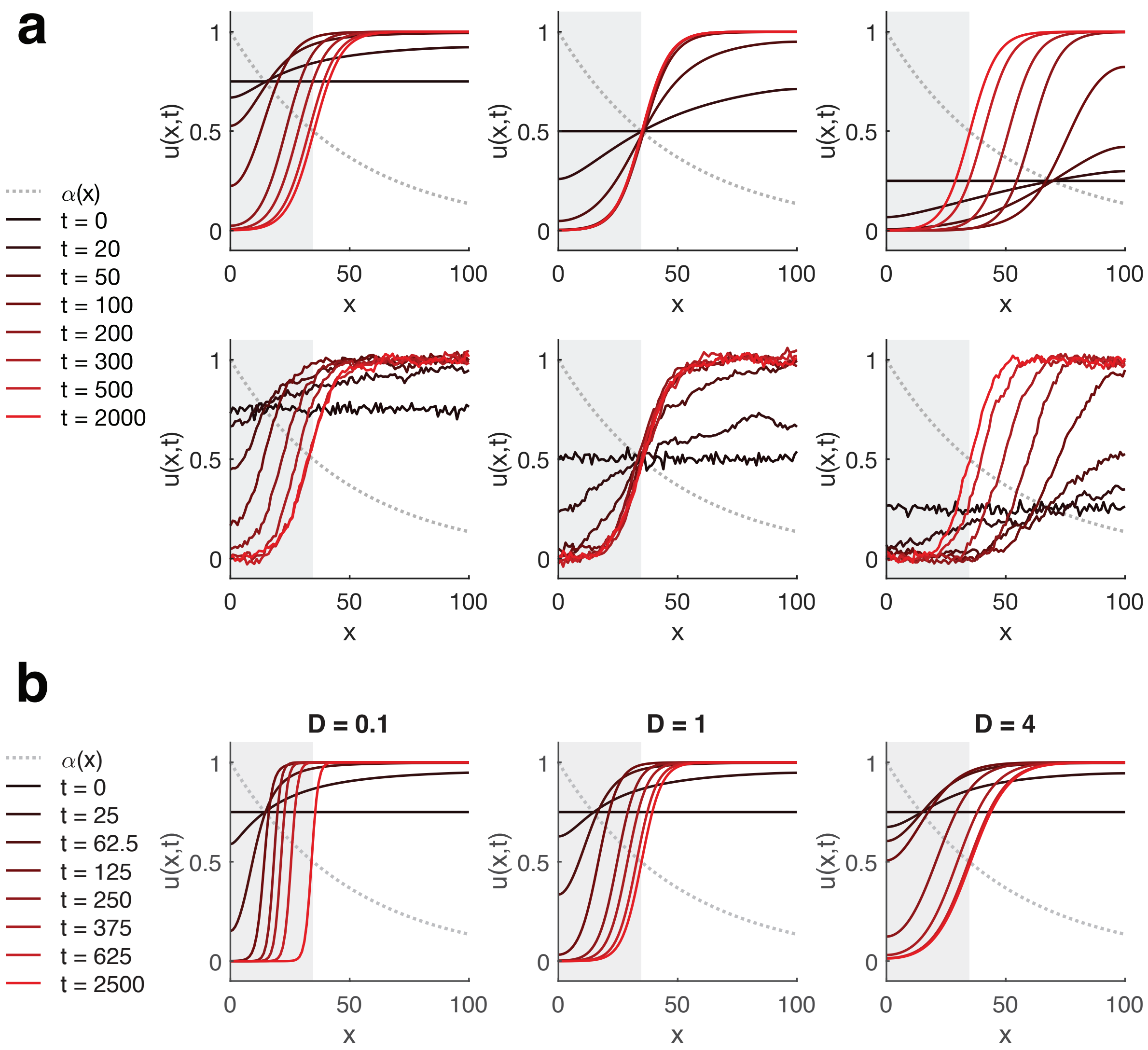

Figure 1: **(a) A near-traveling wavefront may localize to the same point in the domain regardless of uniform-in-space initial condition.** Localized front solutions to a 1D toy model (6) with $a = 0.1$, $D = 2$, and $\alpha(x)$ (gray dotted line) as shown. The value of a homogeneous initial condition (black line, $t = 0$) determines where the front emerges. Subsequent propagation (rightward in gray shaded region, leftward otherwise) causes the front to approach the localization point where the local front velocity is 0 (brighter red indicates later time points). Bottom, small amounts of noise do not disrupt the behavior, as suggested by stochastic simulation of a discretize-space model ($N = 101$ points) with Langevin noise of standard deviation $\sigma = 0.02$ (Euler-Mayurama method, step size $\Delta t = 0.2$). **(b) Reducing D reduces boundary width.** All other parameters are the same as for (a). The local front velocity is independent of D, but in these simulations sharper boundaries appear to approach the localization point more slowly. We hypothesize this phenomenon relates to the dynamics of convergence to front profiles from homogeneous initial conditions.

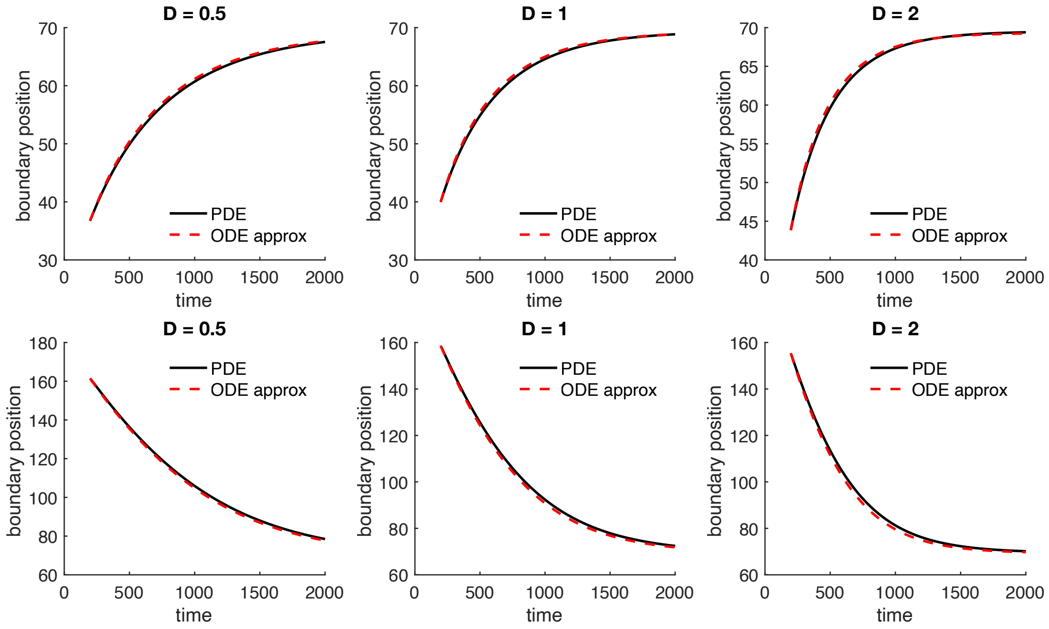

Figure 2: **The analytical solution to an ODE approximation for boundary location closely tracks the boundary position from a PDE simulation.** Solid line is boundary location (crossover point with the unstable steady state) from the PDE simulation; dotted red is the analytical solution (12) to the ODE approximation (10) with $\epsilon = 0$. The only difference between top and bottom plots is the initial condition.

References

[1] Keener JP, Sneyd J. Mathematical Physiology. vol. 8 of Interdisciplinary Applied Mathematics. 2nd ed. Antman SS, Marsden JE, Sirovich L, editors. New York, NY: Springer; 2009.

[2] Zinner B. Existence of traveling wavefront solutions for the discrete Nagumo equation. Journal of Differential Equations. 1992;96:1–27. doi:10.1016/0022-0396(92)90142-A.

[3] Cahn JW, Mallet-Paret J, van Vleck ES. Traveling wave solutions for systems of ODEs on a two-dimensional spatial lattice. SIAM Journal on Applied Mathematics. 1998;59:455–493. doi:10.1137/S0036139996312703.

[4] Chafee N. Asymptotic behavior for solutions of a one-dimensional parabolic equation with homogeneous Neumann boundary conditions. Journal of Differential Equations. 1975;18:111–134. doi:10.1016/0022-0396(75)90084-4.

[5] Casten RG, Holland CJ. Instability results for reaction diffusion equations with Neumann boundary conditions. Journal of Differential Equations. 1978;27:266–273. doi:10.1016/0022-0396(78)90033-5.

[6] Kishimoto K, Weinberger HF. The spatial homogeneity of stable equilibria of some reaction-diffusion systems on convex domains. Journal of Differential Equations. 1985;58:15–21. doi:10.1016/0022-0396(85)90020-8.

[7] Baker RE, Maini PK. Travelling gradients in interacting morphogen systems. Mathematical Biosciences. 2007;209:30–50. doi:10.1016/j.mbs.2007.01.006.

[8] Gomez MM, Arcak M. A Tug-of-War Mechanism for Pattern Formation in a Genetic Network. ACS Synthetic Biology. 2017;6(11):2056–2066. doi:10.1021/acssynbio.7b00077.

[9] Bialecki S, Kazmierczak B, Lipniacki T. Polarization of concave domains by traveling wave pinning. PLoS ONE. 2017;12:e0190372. doi:10.1371/journal.pone.0190372.

[10] Fleming WH. A selection-migration model in population genetics. Journal of Mathematical Biology. 1975;2:219–233. doi:10.1007/BF00277151.

[11] Ikeda H, Ei SI. Front dynamics in heterogeneous diffusive media. Physica D: Nonlinear Phenomena. 2010;239:1637–1649. doi:10.1016/j.physd.2010.04.008.
