# Supplementary material for: Implications of diffusion and time-varying morphogen gradients for the dynamic positioning and precision of bistable gene expression boundaries
Source: PLoS Comput Biol. 2021 Jun 1;17(6):e1008589. doi: 10.1371/journal.pcbi.1008589 (PMC8195430; doi:10.1371/journal.pcbi.1008589)
Supplement: Attachment — Submitted filename: Review.pdf [file pcbi.1008589.s005.pdf]

Review for Dynamic positioning and precision of bistable gene expression boundaries through diffusion and morphogen decay

Morphogen gradients provide critical spatial and temporal information during development. In a number of systems, the spatial information from the morphogen is read-out with remarkable precision. The archetypal example of this is anterior-posterior patterning in the early *Drosophila* embryo. The question of how such precision is achieved has been extensively studied. However, most previous theory has typically ignored dynamic changes in the morphogen. Here, the author explores theoretically potential mechanisms for ensuring such high precision accounting for the underlying dynamics. In particular, they focus on the role of morphogen degradation and diffusion – coupled with a bistable toggle switch - in ensuring precise gene boundary specification. The focus on temporal changes in driving precise readout is very interesting and distinguishes this analysis from previous work. Overall, this works brings new perspectives on how accurate positioning can be achieved in noisy and dynamic systems.

My only major concern is that the approach is only applied to a limited number of scenarios and ignores two cases of particular relevance to Hunchback positioning: (1) scaling of the gene expression boundaries to embryo length; and (2) the interactions between downstream targets driven by the same morphogen (e.g. effects of Kruppel on Hunchback). I outline these further below, with also some minor comments which I believe will improve the manuscript.

Major comments

- As with most similar analysis, the modelling is all performed along a scaled x-axis (e.g. x/L). In lines 299-301 there is a brief discussion of scaling effects, but I feel this is significantly under-developed. This ignores an important element – every embryo is a different size. Recent work (Huang *et al.* ELife 2020) has demonstrated that system size does play a critical role in gene expression boundary positioning in the early *Drosophila* embryo. In particular, Hb can shift position and also robustness is altered in shortened embryos. It would add to the analysis to take the given parameters and explore how boundary specification is altered when only the system size is altered. How robust is the scaling? At what point does the readout "break" (i.e. cross the critical point) and does this correspond to what's observed experimentally in artificially shortened embryos?
- Second, multiple genes are downstream of Bicoid, including Kruppel which is immediately posterior to Hunchback. This is a different scenario to that presented in the paper (of two separate targets with anterior and posterior morphogen inputs). The work of Jaeger has extensively explored these networks. Given a morphogen M, and targets A and B how would the system behave with the following network: M->A , M->B (with different thresholds) and with A and B mutually repressive?

Given the author's framework, these simulations should be relatively straightforward to run. Adding such simulations would add to the paper as they provide further analysis in more realistic scenarios, especially as the author focuses on the early *Drosophila* embryo.

Minor comments

- One mechanism for the refinement of the cell boundary interfaces is mechanical – through differential adhesion, the cells at the boundaries can adjust to sharpen the gene expression profiles. *i.e.* the regulation doesn't have to be directly biochemical, but biomechanical. It would be good to add appropriate detail in the intro [around line 25] about this (e.g. Xiong *et al.* Cell 2013).
- The term "temporal decay" is ill-defined. It took me a while to work out what the author meant (Eq. (5)). This should be more clearly defined as early as possible to improve the understanding. As written, prior to Eq. 5 "temporal decay" could be interpreted as a constant decay rate (so there is continuous decay) or a temporally varying decay rate, or a decrease in the total level of protein.
- There are two ways (at a basic level) to generate a morphogen of the from in Eq. 5: either decay rate increases or production rate decreases. There is evidence for both in the case of Bicoid (e.g. Gregor *et al.* PLoS Biology 2010, Durrieu *et al.* MSB 2017). Does it make any difference to the results what the mechanism is that generates the exp(-\gamma{t}) term?
- Hb should be defined as Hunchback at first usage (it is not defined I think). This will help for the general reader.
- The description of the approximation at lines 107-110 is not especially clear. The important point is (if I'm understanding correctly) that the change in the morphogen is slow compared to the local system response. So, at any particular time point, the morphogen can be taken as static, although on longer time scales it is changing.
- Label axes in the figures directly. It's unhelpful having to go to legend to see what an axis is. Relatedly, I think this is a formatting issue, but the figure quality was very low in the pdf making it hard to perform a detailed assessment of the figures.
- Unclear from the text what the parameter $\bar{b}(x_{crit})$ is – this section needs to be clarified.
