# Supplementary material for: Implications of diffusion and time-varying morphogen gradients for the dynamic positioning and precision of bistable gene expression boundaries
Source: PLoS Comput Biol. 2021 Jun 1;17(6):e1008589. doi: 10.1371/journal.pcbi.1008589 (PMC8195430; doi:10.1371/journal.pcbi.1008589)
Supplement: Attachment — Submitted filename: 2021--03-10 Cover Letter Revisions.pdf [file pcbi.1008589.s006.pdf]

10 March 2021

Dear Dr. Attila Csikász-Nagy and Dr. Florian Markowetz,

Please find the requested revisions for my 9 Dec 2020 submission to *PLoS Computational Biology*, which I have retitled "Dynamic positioning and precision of bistable gene expression boundaries driven by morphogen gradients". I am grateful for the reviewer comments, which were extremely helpful in sharpening my thinking. I believe the manuscript has been substantially improved by addressing reviewer concerns.

The following is a summary of the major changes:

- I have completely rewritten Section 1 to incorporate new analysis of **boundary precision under dynamic morphogen gradients (increasing or decreasing concentration in time) with varying rates of network and gradient evolution, including a metric to assess precision** and further remarks on transient behavior. The original draft only considered decrease in morphogen concentration without examining rate. **Fig 1** has a new subpanel, and **Figs 2 and 3** are entirely new.
- **Estimation of the location of a propagating wavefront using an ODE approximation**. The original draft alluded to local front velocity but did not directly include an ODE formulation or simulations. Plots of the approximation are included in subpanels in **Figs 4 and 5** as well as with an analytical example in Appendix S4.
- **Discussion of the role of diffusion and scaling in the Hb-Bcd model with relation to existing literature**, including implications for experimental observations from Yang et al. (2020) (whence the model was taken). See also new supplementary Fig 3.
- A new subpanel in **Fig 5** addresses the relationship between the diffusivity of two species in a toggle switch and the concentration of morphogen at which a propagating wavefront will localize. Further discussion in this section discusses the transient behavior and robustness of the design.
- In light of the substantial revisions and to clarify my contributions, I have **reframed the work to emphasize mathematical approaches for conceptualizing dynamic positional information, and design tradeoffs among accuracy, precision, placement, timing, and diffusion**. Reframing is most evident in the introduction, abstract, author summary, and beginning of the discussion section.

I have attached the responses to all reviewer comments, grouped by subject/concern, with my responses in blue.

Sincerely,

Melinda Liu Perkins

PRECISION (NO DIFFUSION)

Initial conditions

(Reviewer 1) The author also uses an arbitrary initial condition in which each cell shows a random gene expression. It is not clear to me that this is biologically relevant. In the current scenario cells are kept in a noisy naive state despite being already in the presence of the gradient. A more realistic scenario would include the fact that the cells are already in that gradient by either assuming that each cell is in equilibrium with the gradient before its decay start or by including gradient formation in the formulation of the problem.

Thank you for the suggestion. Noisiness in initial conditions remains a challenge for boundary precision even if the network is acting simultaneously with gradient emergence. I have now analyzed this situation in the revised Section 1 and especially Fig 2, where I observe that in general, if network dynamics are sufficiently fast relative to the rate of gradient formation, the effect of initial conditions on precision is minimal. I have also added a paragraph in the discussion (lines 577-588) concerning stochasticity during dynamic evolution.

In the process of addressing this comment I have also significantly extended the discussion of time-varying gradients to explicitly incorporate initial conditions when assessing precision enhancement. In principle the framework also handles structured initial conditions, such as might arise when one network temporally assumes control from the output of a previous network.

Quantitative role of decay rates

Limiting case (control): no decay

(Reviewer 1) In the section "Enhanced boundary precision through temporal gradient decay" the author proposes that initial condition variability is reduced by the gradient decay. In order to check the validity of this, the author should show a comparison of the pattern formation with and without gradient decay to explore the differences.

(Reviewer 3) The author needs to show more details on the role of the morphogen decay in the refinement of the boundary in the toggle-switch model shown in Figure 1. The repression of u2 on u1 could also help to refine the boundary as the concentration of u2 increases. Hence it is necessary to show the boundary evolution without morphogen decay as a control.

I have incorporated the time lapse for the control case into the new Fig 2 on the far right column. Note that this section has been significantly restructured to emphasize gradient emergence first, followed by the hypothesized precision that might result from gradient decay, but the control is the same in both cases.

Varying decay rate

(Reviewer 1) I found interesting the analysis of the gradient decay from a bifurcation point of view. Nevertheless in general the same effect would be true for any other varying parameter of the gene regulatory network.

In principle the observed effects on precision could indeed be observed by time variation in parameters other than the morphogen, provided that the parameter is monotonic in both time and space over some scale of interest. I have made note of this observation in lines 136-139.

(Reviewer 1) If the author wants to focus exclusively on the the decay of the gradient, a more detailed analysis of the biological timescales is required e.g. is the decay of the morphogen required to be slower than the decay of the GRN transcription factors? What happens with the boundary when this timescale separation does not hold?
(Reviewer 3) It also would be better that the author could run some parameter sensitivity analysis to show the robustness of the conclusion, e.g., vary the decay rate of the morphogen.
These are good suggestions. In the revised Section 1 I have expanded the analysis to a range of relative timescales between the temporal rate of morphogen evolution and the convergence rate of the genetic network to a steady state, which relies on a combination of the network parameters. In general, static or relatively fast gradients produce less precise results (new Figs 2 and 3).

(Reviewer 1) In the same section, one of the limitations of the mechanism proposed is that once the gradient decays completely, the boundary will completely disappear (there will only be a monostable u_2 regime). What are the time limit constrains for cell fate decision in this aspect?
This is true. Maintaining the boundary requires either maintaining the gradient (e.g., by decreasing morphogen concentration through increased protein degradation rather than decreased production [Durrieu et al. 2018]), or, as the reviewer has suggested, by rendering a cell fate decision (making a readout) before the gradient has disappeared. The amount of time to take a readout that will still result in a boundary is absolute upper bounded by the time at which the posterior end of the bistable region reaches the front of the embryo, which is an extreme case. A lower bound on time to readout can be estimated based on the relative rates of gradient and system evolution and a target precision level; see revised section 1 and new Fig 3.

(Reviewer 2) There are two ways (at a basic level) to generate a morphogen of the from in Eq. 5: either decay rate increases or production rate decreases. There is evidence for both in the case of Bicoid (e.g. Gregor et al. PLoS Biology 2010, Durrieu et al. MSB 2017). Does it make any difference to the results what the mechanism is that generates the exp(-\gamma{t}) term?
Thank you for bringing these sources to my attention. From Durrieu et al. (2018) Fig. 6A it appears that whether temporal decrease in morphogen concentration is driven primarily by an increase in degradation rate or a decrease in production rate would primarily affect the rate at which the gradient flattens, with decreased production resulting in faster flattening, and increased degradation retaining a gradient-like profile even as concentration levels decrease. The main implications of the mechanism for the model in this manuscript would pertain to how a cell fate decision (readout) is made. If production is decreased, then a readout must be made before the gradient is entirely flat at 0 concentration (at which point the entire domain would be monostable for low u1). If degradation is increased, the longer-persisting gradient profile should reduce the rate at which the bistable region shifts anteriorly and therefore allow more time for a readout to be taken. I have addressed this point in lines 219-226.

PROPAGATING WAVEFRONT (DIFFUSION)
Later gene networks (e.g., GAP)
(Reviewer 1) The application fo the Bicoid-Hunchback system was interesting. The author dissected the role that diffusion and gradient decay have in this particular system. Nevertheless, in its current state is just an analysis on a toy-model formulated in a different paper and it was not clear the biological impact of the results. For instance the author analyses what happens at times greater than 60 minutes in the static gradient case (thin lines in Fig 2a,b) but it does not determine what timepoints were used for these lines or why it is relevant in the fly patterning that the boundary stops moving. In particular we know that boundaries in the fly move while they pattern as a result of the GRN dynamics of the GAP genes.
I have completely reframed this section to emphasize the intended contribution. Specifically, I developed a mathematical perspective for looking at bistable gene expression boundaries with diffusion as propagating wavefronts with velocity that varies in space, and established that an ODE approximation based on local front velocity can make good quantitative approximations for the location of a boundary in a full PDE. I used the Bcd-Hb model from the literature as an example of a system that can be analyzed as a propagating wavefront, which is more a mathematical point than a biological one. Therefore my intent was not necessarily to make new statements about the relevance of Yang et al.'s model to the mechanisms at play in the actual fly, so much as to motivate the relevance of a different kind of mathematical intuition for understanding the results of certain classes of biological models. That said, in the new draft I have included comments about the relevance of diffusion in this system (lines 431-447) as inspired by the consideration of this system in light of the behavior of propagating wavefronts.

(Reviewer 2) Second, multiple genes are downstream of Bicoid, including Kruppel which is immediately posterior to Hunchback. This is a different scenario to that presented in the paper (of two separate targets with anterior and posterior morphogen inputs). The work of Jaeger has extensively explored these networks. Given a morphogen M, and targets A and B how would the system behave with the following network: M->A, M->B (with different thresholds) and with A and B mutually repressive?
Provided that the resulting system were bistable in some regime, then the front propagation perspective would lend itself to the analysis. While it is interesting, I am not convinced the addition of this model to the text would serve to emphasize the intended contribution of this work, which I hope has been clarified in the new draft.

(Reviewer 3) It is interesting to see the Hb boundary keeps shifting after 60 min into nuclear cycle (nc) 14 in the simplified model shown in Figure 2. But the author needs to point out that such a phenomenon is not biological relevant. As it is well known that the Hb expression is driven by the Bcd activation and self-activation (P2 enhancer) only before early nc14. Its

regulation is switched to the stripe enhancer without Bcd activation and self-activation but repression from other gap genes such as Kni and Kr.
The reviewer is correct that this is not biologically relevant, and I have acknowledged this point in line 421, alongside the observation that even a one-minute delay in readout would only be expected to incur an additional error 1 less than 1 micrometer. Nevertheless, as a mathematical point of interest I think the propagation is still interesting—if nothing else as a theoretical argument that autoactivating Hb alone upon a Bcd gradient is insufficient to produce a stable boundary without other involved factors.

Importance of initial conditions
(Reviewer 1) Similarly, it looks that the initial condition is very important in the positioning of the boundary. Nevertheless I am not sure to which extent the proposed initial conditions are an artefact used in the toy model proposed in [37]. In this aspect I agree that the author has just replicated the same conditions, but then I missed further exploration of this property.
It is true that the initial conditions affect where a boundary arises. I have attempted to discuss some of the qualitative metrics associated with boundary emergence in the text. Once it is known where a front is located, then provided it is in a bistable region, the local front velocity well predicts where it will propagate in an arbitrary timespan.

Please see the second example in this section for more exploration on the role of initial conditions and local front velocity on boundary placement.

(Reviewer 1) On the same note, in the last section of the paper (Fig. 3). The author requires that the initial levels of the u1,u2 are intermediate levels of expression. Similarly to the initial questions, does this imply that there was no cross-repression until the start of the simulation? What happens when the levels are already in equilibrium with the gradient? What happens if they are not in equilibrium of the gradient but then they follow gradient formation?
The results do not require that u1 and u2 begin with uniform intermediate levels of expression. I used them to demonstrate the point that boundaries can spontaneously emerge from uniform conditions in systems with spatially varying bifurcation parameters, unlike in systems with spatially homogeneous bifurcation parameters, BUT that with the proposed design a boundary can still be accurately placed in a domain regardless of starting point. I admit this point may be more relevant to synthetic than to developmental biologists.

If "the levels are already in equilibrium with the gradient" then the levels track either only the high state or only the low state throughout the domain, or else a boundary exists where the front velocity is zero. Regarding boundary dynamics as the gradient emerges, while this is no doubt an interesting point, I'm not convinced that it would illustrate the primary contribution of this section, which I hope is clearer in the revision.

(Reviewer 4) The diffusion mechanism seems fragile to me; slight changes in initial conditions appear to stongly affect the dynamics of boundary formation (Figure 3a). Since patterns are often continuously interpreted by downstream genes, this can have profound effects on the developmental process. I guess the issue is that in development most patterns are built on top of already existing patterns, whilst here the point is made that patterns even arise from uniformly expressed genes. So some asymmetry in the initial distribution of u1 and u2 (a pre-pattern) should make the dynamics very stable. Is that correct? This point is probably worth a paragraph in the manuscript.
Yes, this is indeed correct. I have incorporated this discussion into lines 516-530.

Scaling effects
(Reviewer 2) As with most similar analysis, the modelling is all performed along a scaled x-axis (e.g. x/L). In lines 299-301 there is a brief discussion of scaling effects, but I feel this is significantly under-developed. This ignores an important element – every embryo is a different size. Recent work (Huang et al. ELife 2020) has demonstrated that system size does play a critical role in gene expression boundary positioning in the early Drosophila embryo. In particular, Hb can shift position and also robustness is altered in shortened embryos. It would add to the analysis to take the given parameters and explore how boundary specification is altered when only the system size is altered. How robust is the scaling? At what point does the readout "break" (i.e. cross the critical point) and does this correspond to what's observed experimentally in artificially shortened embryos?
I thank the reviewer for bringing my attention to this interesting work. I have added the following paragraph (lines 448-466) and Supplementary Fig 3 in relation to this point:

Finally, in the example shown here, boundary placement for propagating wavefronts behaves rather similarly to a traditional thresholding model with respect to embryonic scaling; that is, in the absence of other adjustments, changing embryo length without altering the absolute scale of the gradient should negligibly affect the absolute position of the boundary and therefore produce an anterior or posterior shift with respect to normalized coordinates, depending upon whether the embryo were lengthened or shortened. In practice, however, gradients may change shape with embryo geometry. For example, experiments conducted in *D. melanogaster* with artificially shortened embryos produced Bcd gradients later and lower in amplitude than WT, resulting in a posterior shift in the Hb boundary of just under 4% percent embryo length [Huang et al 2020], as compared to the approximately 12% that would be otherwise predicted for embryos 80% the length of WT. Even if gradients were to scale perfectly, our simulations indicate modest effects on boundary placement when initial conditions are not also scaled. With the dynamic gradient, the additional shift is less than 0.5% for embryos at 80% WT length, but rises to almost 2% for embryos 50% WT length (Fig S3), at which point cells within the bistable region begin with nonzero Hb concentration. Further research would be necessary to determine whether very large or small embryos suffer from robustness issues due to compounding errors, as well as whether initial conditions of fixed length scale have evolved to be compatible with a range of scales downstream.

Fragility of mechanism
(Reviewer 3) it is also necessary to do the parameter sensitivity analysis (especially on the diffusion constant) on the role of diffusion in pattern refinement shown in Figure 3. In the gap

gene network, it is often believed that the diffusion role is very limited. So it would be better that the author could discuss some biological relevance.
I have added some analysis of the diffusion constant to Fig 5b. Regarding the relevance of diffusion, I have added the following paragraph (lines 431-447) to the discussion of the Bcd model:

Viewing the Hb boundary as a propagating wavefront suggests a straightforward mechanism for stabilizing the pattern---namely, to reduce protein diffusivity to 0. Experimental measurements indicate that effective diffusivity, or internuclear coupling strength, begins to decrease 20 min into nc14, reaching 0 by 30 min [Bothma et al 2018]. This time window coincides with the time the boundary reaches the bistable region in the WT embryo in our simulations. It is worth noting that 20 min also marks the start of a reduction in slope in the data for *stau-* reported in Fig 6 of [Yang et al 2020], while 30 min is the point at which their model predictions for Bcd1.0 boundary placement also cease to recapitulate experimental results. In the WT simulations, the Hb boundary at 20 and 30 min for a static gradient is nearly identical to the location of the boundary for the system with a dynamic gradient at 20, 30, and 50 min into nc14 (Fig S3). Temporal decrease in morphogen concentration after the loss of diffusion would not be expected to change boundary position, since cells to the anterior of the boundary would maintain their high state as the underlying region became locally bistable. It is therefore worth considering to what extent the gradient dynamics are in fact transmitting new dynamic positional information, as opposed to being primarily symptomatic of a full handoff in control of the Hb boundary to downstream networks [Jaeger 2011].

Otherwise, the reviewer is suggesting a philosophical point I hope to reiterate, namely, that systems with and without diffusion are mathematically distinct and admit different classes of behaviors, and that a tradeoff between precision and placement may serve as a design consideration of sorts for bistable patterning systems.

PRESENTATION
(Reviewer 1) In the second part of the manuscript "Boundary shifts induced by near-traveling fronts". It is not clear to what is the benefit of the mathematical formulation. I expected to see some applications of formula (9) in the manuscript. If I understood correctly, all the analysis is done by performing numeric integrations of formula (8), and in particular the same exact formulas than the ones presented in [37].
It may not have been clear in the original draft that the analysis is not restricted to the formulas presented in [37], as it is applied to an entirely separate example unique to this paper in which a boundary forms over two antiparallel morphogen gradients that guarantee the boundary stalls at a particular location within the embryo (old Fig. 3, new Fig. 5) as well as a demonstration of front localization at multiple points in a domain (old Fig. 4, new Fig. 6). Formula (9) was applied to generate the local front velocity plots in old Fig. 2 (new Fig. 4), and in the new draft has been explicitly applied to the ODE simulations in Figs. 4 and 5 as well as the nullcline plot in Fig 5b. Some analytical results for a 1D toy model are also available in the S4 Appendix.

Since (9) can be calculated from concentration alone, it also serves as an intuitive tool for prediction or synthetic design; for example, one can ascertain which concentration of morphogen results in 0 local front velocity and thereby ascertain which direction a front will propagate when presented with higher or lower concentration than this “critical” concentration, regardless of the

exact shape of the morphogen gradient. This was indeed the procedure I used to construct the examples in old Figs. 3 and 4 (now 5 and 6).

(Reviewer 1) lines 400-403 "Thus, for a fixed uniform initial condition, the apparent threshold at which cells are activated will shift, giving rise to the observed inaccuracy in boundary placement in such models.". I do not think that the word "inaccuracy" is accurate. We know that boundaries move in many embryonic systems. The idealisation that boundaries must be static is an artifact of our imposition of the French flag model.

By “in such models” I indeed meant to refer to the imposed French flag model, which I contrast with dynamic position information models that incorporate empirical observations of shifts in embryonic systems. I have clarified this statement accordingly.

(Reviewer 1) Whilst it looked interesting, I could not fully follow the argument of paragraph 414-428 in the discussion.

I have rewritten this point using an autoregulatory element as an example. Basically, a stable steady state is only accessible from initial conditions in the basin of attraction for that state. So, if a signal establishes the bistability of a network but some other upstream component establishes the initial conditions, then disrupting the signal’s ability to regulate OR disrupting the upstream component could cause the system to appear unresponsive to the signal (end up in the low state), but through different means. Specifically, disrupting the upstream component means the system can never have initial conditions that place it in the basin of attraction for any state other than a default. Disrupting the signal’s ability to regulate means the appropriate stable steady states never exist in the first place:

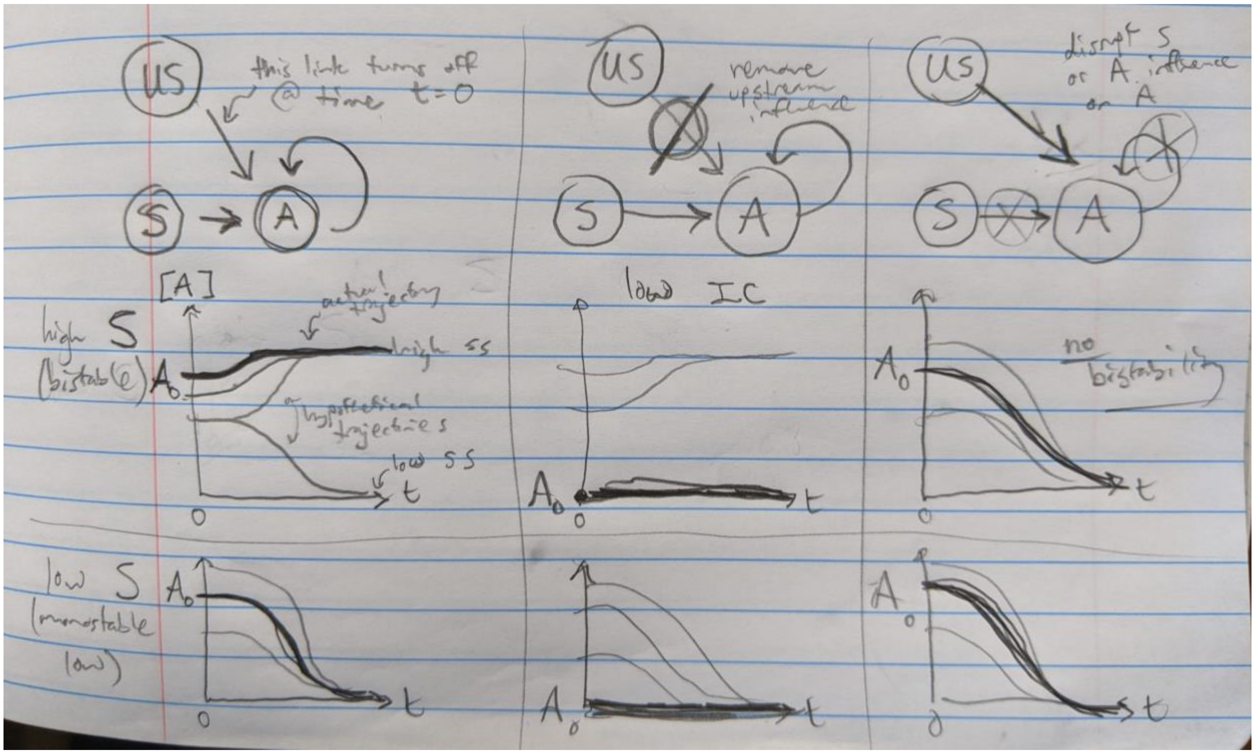

(Reviewer 3) Temporal refinement in gene expression domains has been investigated with the gene circuit model of gap genes, e.g., Manu et al., PLos Bio, 2009. So the first sentence in the Abstract is not accurate.
Apologies for the misunderstanding; by “traditional models” I more accurately meant “French flag models”. I consider Manu et al. to fall under recent work amenable to analysis in the language of dynamic positional information. I have rewritten the Abstract as well as parts of the discussion to clarify.

(Reviewer 4) I would emphasize the differences between the two ideas in terms of what they can do. I am mainly thinking about how the first has "the boundary shifting up the gradient" and the second tends to shift "down the gradient". It would make the article stronger if the author presents two orthogonal ideas, instead of 2 variants on a single idea. OR perhaps I misunderstood and both are perfectly reversible? If so, that is worth elaborating on.
I hope I have addressed this concern in my reframing of the paper to emphasize conceptual mathematical tools, which can be applied to multiple models including the two proposed mechanisms.

(Reviewer 4) Regarding the second part on diffusion and moving boundaries, I would think the manuscript needs a better argumentation for novelty. For instance, the paragraph on wave pinning (now on page 10) could be moved forward to better provide a contrast between the author's work and existing literature. (Just a suggestion!) Also, some relevant literature was

missed (understandably, it is not easy to find): "Having the right proportions: Interacting interfaces ensure robust spatial patterning" Vakulenko et al., Physical review letters 103 (16), 2009.
I appreciate the suggestion and have adopted it. I also thank the reviewer for bringing my attention to this paper, which I have cited in lines 303-306:

The approach is similar in spirit to previous work suggesting that expression domains can be predicted to expand or shrink based on terms arising from local field approximations [Vakulenko et al 2009], but the mathematical details and conceptual treatment differ.

Minor comments
(Reviewer 1)
- line 105 is not clear what the author means by "cell behaviour can be local", or "cell behaviour can be instantaneous". This has been clarified in lines 125-128.
- line 146 "The speed and direction of front propagation depend on parameter values, specifically on which of the two stable steady states is more favourable". It is not clear what a "more favourable" state means or how it is related to the parameter values mentioned in the first part of the sentence I have clarified that "more favorable" refers to which of the steady states has a larger basin of attraction.
- line 196 "ambryo" should be "embryo" Fixed.
- lines 163-169 the definition of 'near-travelling' front could be a bit clearer I have revised the terminology to refer simply to "propagating fronts". The motivation behind a different terminology is that traveling fronts propagate on domains in which bifurcation parameters are homogeneous, whereas in the models I consider bifurcation parameters vary in space along the propagation domain. Mathematically the distinction exists because traveling fronts can be solved for by changing coordinates to a moving frame of reference, which is not possible if the spatial bifurcation parameters are not homogeneous.

(Reviewer 2)
- One mechanism for the refinement of the cell boundary interfaces is mechanical – through differential adhesion, the cells at the boundaries can adjust to sharpen the gene expression profiles. i.e. the regulation doesn't have to be directly biochemical, but biomechanical. It would be good to add appropriate detail in the intro [around line 25] about this (e.g. Xiong et al. Cell 2013). I have added some citations for biomechanical regulation in the indicated part of the introduction.
- The term "temporal decay" is ill-defined. It took me a while to work out what the author meant (Eq. (5)). This should be more clearly defined as early as possible to improve the understanding. As written, prior to Eq. 5 "temporal decay" could be interpreted as a constant decay rate (so there is continuous decay) or a temporally varying decay rate, or a decrease in the total level of protein. I have replaced all instances of the term "temporal decay" with "temporal decrease [in morphogen concentration]".
- Hb should be defined as Hunchback at first usage (it is not defined I think). This will help for the general reader. I have added this definition.

- The description of the approximation at lines 107-110 is not especially clear. The important point is (if I'm understanding correctly) that the change in the morphogen is slow compared to the local system response. So, at any particular time point, the morphogen can be taken as static, although on longer time scales it is changing. This explanation has been rewritten.
- Label axes in the figures directly. It's unhelpful having to go to legend to see what an axis is. Relatedly, I think this is a formatting issue, but the figure quality was very low in the pdf making it hard to perform a detailed assessment of the figures. I have added axis labels and clearer legends.
- Unclear from the text what the parameter *b* "(*x*!"#$) is – this section needs to be clarified. This section has been rewritten.

(Reviewer 4)
- The liberal use of quotation marks to mark special words, breaks my reading flow. I would in many cases simply remove them. I agree the usage was excessive and have removed most occurrences.
- Fig 1b: the gray gradients are difficult to compare. Could you plot them all in each graph, but the focal one darker and the other light-gray? I have implemented this suggestion in the new Fig. 3.
- Fig 2: why do the red and golden curves have a bit of a bump just before the gradient? The bump results from the fact that the high steady state is slightly higher in the bistable regime than in the monostable regime, as a consequence of the choice of regulatory function f(b,h).
- Fig 2: it is not always clear which color is u1/u2. Repeat the legend of fig 1? Also the little network of Bcd and Hb could have colors corresponding with the graph below. I've added a legend to clarify.
- Fig 3a: a legend for the temporal color code is missing. (dark gold to light gold) I've added a legend.
