# Supplementary material for: Implications of diffusion and time-varying morphogen gradients for the dynamic positioning and precision of bistable gene expression boundaries
Source: PLoS Comput Biol. 2021 Jun 1;17(6):e1008589. doi: 10.1371/journal.pcbi.1008589 (PMC8195430; doi:10.1371/journal.pcbi.1008589)
Supplement: Attachment — Submitted filename: 2021--04-27 Response to Reviewer Comments.pdf [file pcbi.1008589.s007.pdf]

5 May 2021

Dear Dr. Attila Csikász-Nagy and Dr. Florian Markowetz,

Please find the requested revisions for my 9 Dec 2020 submission to *PLoS Computational Biology*, which I have retitled "Implications of diffusion and time-varying morphogen gradients for the dynamic positioning and precision of bistable gene expression boundaries". The changes are as follows:

- The work has been reframed to more explicitly tie together the two main results, including updates to the Abstract, Author Summary, Introduction, and Discussion sections.
- The link between boundary placement and precision in systems lacking diffusion has been made more explicit in the Results. This point relates intimately to the matter of variation in initial conditions, which have received further mention in the Discussion.
- The relation of this work to existing theoretical and experimental work in the literature has been elaborated upon in the Introduction and Discussion, including the addition of reference [39].
- The choice of a deterministic model has been stated more clearly upfront.

I have attached the reviewer comments with my responses in blue.

Thank you for your consideration.

Sincerely,

Melinda Liu Perkins

Reviewer #1: Even though the author has worked hard into creating a new version of the manuscript (it actually looks like a fully new manuscript), I still hold the same main concerns I posed in the first version. The paper is formed by two disjointed parts that do not provide deep enough - biologically relevant - analysis by themselves. I still believe that the author should split the article in two and address the biological problems/limitations that barely mentions in the discussion.

While I respect the reviewer's concerns, I believe that we have different expectations for the nature of this work. I have chosen an approach that starts from a simple mathematical model and pushes to its logical conclusion. In doing so, I acknowledge and accept that I may examine some "biologically irrelevant" directions, from the standpoint of their having not (yet) been experimentally observed. Nevertheless, many disciplines have a long tradition of playing with toy models or conducting thought experiments for the purpose of developing intuition.

Returning to this work specifically, I have rewritten the introduction and abstract to clarify why, to my mind, the ideas are not disjoint. Essentially, models with and without diffusion lend themselves to different kinds of mathematical thinking and analysis, even if the biological outcomes appear similar (e.g., because quantitative data fitting can reproduce the results with or without diffusion). This is an important point for those who intend to get mechanistic insight out of a model, as well as for those who wish to approach biology from the standpoint of a designer. In the specific situations here, I demonstrate that these differences lead to different modes of pattern refinement and imply different ways of interpreting dynamic positional information conferred by morphogen concentration. I do not claim to make any definitive statements about whether specific living systems ought to be modeled one way or the other, nor do I claim to have exhaustively explored the properties of the models chosen here. I simply aim to add a different perspective to the conversation on this topic.

On that note, in response to the reviewer's concern about biological problems/limitations I have revised the discussion to more clearly address the challenges of mapping models to biological systems (lines 629-642).

In short, the manuscript illustrates that simple, general models can admit interesting patterning behaviors, and that the perspective from which a model is analyzed shapes the intuitive process of understanding, making predictions, and generating new designs/hypotheses. Naturally I would be pleased if explicit experimental examples of the hypotheses were identified or implemented synthetically. Regardless, I still believe the ideas proposed here have a use in addressing philosophical points and stimulating thinking among those inclined to apply dynamical systems approaches to their work.

In the same line, the new title of the paper seems too generic, it does not address the main two topics that the paper tackles 1) dynamic morphogen (different to "dynamic positioning") and 2) diffusion of gene products.

I agree. The paper has been retitled "Implications of diffusion and time-varying morphogen gradients for the dynamic positioning and precision of bistable gene expression boundaries".

1) On one hand the use of arbitrary random initial conditions while ignoring noise in the evolution. If the evolution of the systems is considered to be deterministic, where does the noise in initial conditions come from? Without a more dedicated analysis, it looks like the author is cherry-picking the biological conditions of the system. This should not only be mentioned in the discussion, but should be addressed in the introduction, and also tested along the manuscript. The author is aware of this since makes references to papers that have addressed these issues in the past, so I found misleading that this was not addressed properly along the text. For instance before starting to present their theory, the authors should make clear what other approaches have been done in the past regarding moving boundaries (e.g. refs [15] and [29]), and what place does their study plays in it.

I now reiterate upfront that this work only considers deterministic models (line 53), the shortcomings of which are discussed in lines 643-647. I also mention that empirical work would be necessary to verify how important initial variation might be (lines 651-653).

The initial conditions do not have to be random to apply the methodology, as is now more clearly stated in the text (lines 332-335). I chose random initial conditions for the first example because they cleanly illustrate a point, though I think it is at least plausible that some systems may have more variation in initial conditions than in subsequent expression levels, e.g., if upstream networks are less tightly regulated than downstream ones (lines 181-183).

I have revised the introduction and discussion to clarify the relation of this work to the indicated references (lines 41-52 and 629-642). I have also made explicit the connection between placement and precision in systems without diffusion; specifically, the variable initial conditions in the plots do not have to represent individual cells, but can also represent embryo-to-embryo variation in uniform starting concentrations, and the metric still applies as described (lines 165-177).

2) The PDE/ODE travelling fronts still lacks an explicit connection between theory and results. In the text is not clear at all the connection between (13) and (14) or how it is applied in the Hb-Bcd case. There is a bit more of insight in the Appendix, but still is very hard to follow, or to understand what exactly do we learn out of it or how should we apply it. This would be more clear in an individual manuscript where the motivation, previous work, and results are well structured.

As explained in the text, solving (13) for different concentrations of morphogen gives c(x), which appears in equation (14). The central insight is that morphogen concentration determines the propagation velocity (speed and direction) of a bistable gene expression boundary, and that knowing the relationship between concentration and velocity allows one to predict how a boundary will shift over an arbitrary morphogen profile (position vs. concentration), either through intuitive reckoning or an ODE approximation.
